# High Speed Stereovision Setup for Position and Motion Estimation of Fertilizer Particles Leaving a Centrifugal Spreader

**DOI:** 10.3390/s141121466

**Published:** 2014-11-13

**Authors:** Bilal Hijazi, Simon Cool, Jürgen Vangeyte, Koen C. Mertens, Frédéric Cointault, Michel Paindavoine, Jan G. Pieters

**Affiliations:** 1 Institute of Agricultural and Fisheries Research, Burg. van Gansberghelaan 115, Merelbeke 9820, Belgium; E-Mails: hij.bilal@gmail.com (B.H.); Jurgen.Vangeyte@ilvo.vlaanderen.be (J.V.); Koen.Mertens@ilvo.vlaanderen.be (K.C.M.); 2 Institut National Supérieur des Sciences Agronomiques, de l’Alimentation et de l’Environnement, boulevard du Docteur Petitjean 26, Dijon 21079, France; E-Mail: frederic.cointault@agrosupdijon.fr; 3 Laboratoire d’Etude de l’Apprentissage et du Developpement, University of Burgundy, Esplanade Erasme 11, Dijon 21000, France; E-Mail: Michel.Paindavoine@u-bourgogne.fr; 4 Department of Biosystems Engineering, Ghent University, Coupure links 653, Gent 9000, Belgium; E-Mail: Jan.Pieters@Ugent.be

**Keywords:** stereovision, motion estimation, fertilizer, centrifugal spreader

## Abstract

A 3D imaging technique using a high speed binocular stereovision system was developed in combination with corresponding image processing algorithms for accurate determination of the parameters of particles leaving the spinning disks of centrifugal fertilizer spreaders. Validation of the stereo-matching algorithm using a virtual 3D stereovision simulator indicated an error of less than 2 pixels for 90% of the particles. The setup was validated using the cylindrical spread pattern of an experimental spreader. A 2D correlation coefficient of 90% and a Relative Error of 27% was found between the experimental results and the (simulated) spread pattern obtained with the developed setup. In combination with a ballistic flight model, the developed image acquisition and processing algorithms can enable fast determination and evaluation of the spread pattern which can be used as a tool for spreader design and precise machine calibration.

## 1. Introduction

Over the past 60 years the use of mineral fertilizers has allowed farmers to drastically increase their crop yields. Mineral fertilizer application currently accounts for 43% of the nutrients that global crop production extracts each year, and the contribution may be as high as 84% in the years to come [1]. Organic nutrient sources are unlikely to challenge mineral fertilizer in the future [1]. The majority of farmers use a broadcast spinner spreader, also known as centrifugal spreader, because of their large working width, low cost, robustness and spreading efficiency. The working principle is simple: the fertilizer particles fall onto a disk equipped with vanes. The disk rotates at 400 to 1000 rpm and consequently throws the particles into the field. Nowadays, fertilizer spreaders are equipped with two disks rotating in opposite directions, enabling an easier operation with larger working width and reduced number of tramlines.

Several factors affect the fertilizer distribution in the field such as the spreader settings and the fertilizers physical properties. This distribution or spreading pattern should correspond to the crop’s needs as closely as possible. In fact, applying an imprecise amount of fertilizer might actually decrease the production efficiency [2–6]. For example, lodging of cereals due to an excess of nitrogen input decreases profit substantially [7]. To be able to spread the correct amount of fertilizer on the right place in the field, correct spreader settings are determined by performing a calibration test taking into account both machine and fertilizer properties. In most cases the fertilizer particles are collected in standardized trays and then weighed [8]. Because this is a long and fastidious method, several alternative techniques have been developed to characterize the spreading process more efficiently and calibrate spreaders [2,4,9–16]. Those hybrid techniques first determine the fertilizer particles’ ejection parameters (direction, speed and in some cases also the size) and secondly use a ballistic flight model to estimate their landing points in the field [17]. Among those methods, the ones using an imaging system are the most promising because they offer the possibility to measure all ejection parameters without interfering in the fertilizer ejection flow [18]. In [19] a new method was developed to estimate the motion of the fertilizer particles inspired from the Particle Image Velocimetry (PIV) motion estimation techniques [20]. This method gave accurate results. However, because it is based on a 2D imaging system, the method can only be applied for particles moving parallel to the image sensor. In practice, fertilizer particles are generally ejected with a vertical angle that can reach up to 15° with the horizontal plane [15] and even with a flat disk, vertical angles up to 4.18° are found [16]. Hence a 3D approach would increase the accuracy of the motion estimation for particles leaving the spinning disk of fertilizer spreaders. The objective of this paper was to develop such a 3D system for automated position and motion estimation of fertilizer particles leaving flat disks and more important also for inclined spreaders or spreaders with conical disks.

In combination with a ballistic flight model this system can be used by manufacturers as a tool for spreader design because the prediction of the spread pattern allows a fast inspection of the effect of spreader settings. The system has potential for affordable spreader calibration at farm level and as online feedback sensor on modern fertilizer spreaders.

## 2. Experimental Section

The developed system consists of two main components. The hardware part is formed by the imaging system (IS): the high speed binocular stereovision setup. The software consists of a 3D position and motion estimation algorithm which combines a segmentation algorithm with a stereo and time matching algorithm. All image processing has been done with Matlab (version 2013a) (Natick, MA, USA).

### 2.1. High Speed Binocular Stereovision Setup

The design of the IS highly depends on several characteristics of the spreading process: the speed of event occurrence, scale of the event to be monitored and the size of the objects. The speed of event occurrence is related to the velocity of the fertilizer particles. This dictates the allowable exposure time, the frame rate and the field of view of the cameras. An exposure time that is relatively long in relation to the particle velocity results in blurred images. When the frame rate is too low, ejected particles will be out of the field of view before a sufficient number of images is taken. During spreading, fertilizer particles are ejected with velocities of up to 40 ms^−1^ [18]. The field of view is dependent on the velocity of the particles, but also on the properties of the lenses and the height of the camera setup. Furthermore, it is necessary that all particles of a throw, *i.e.*, a group of particles ejected by the same vane during one revolution of the disk, have left this vane and are in the field of view of both cameras.

The amount of detail necessary (resolution per particle) is dependent on the size of the particles. This can vary between 1 and 5 mm with a distribution dependent on the fertilizer type. The particle resolution is determined by the field of view and the resolution of the individual camera sensors. To obtain enough detail, this parameter should be maximized.

To fulfill the practical conditions mentioned above, a high speed imaging system (HSIS) with a minimum rate of 500 frames per second, a field of view of around 1 m^2^ and a pixel/mm ratio inferior or equal to one was proposed. Two monochrome high speed cameras (HS-3, N3, Integrated Design Tools, Tallahassee, FL, USA) equipped with two Xenoplan lenses (Schneider-Kreuznach, Bad Kreuznach, Germany) were used. The HS-3 and N3 cameras have a resolution of 1280 × 1024 pixels^2^, a frame rate of 1000 frames/s at full resolution and a pixel size of 12 × 10^−6^ m × 12 × 10^−6^ m. The lenses had a focal length of 28 mm and f-stop of 2. An exposure time of 100 µs was used in combination with sufficient lighting. The two cameras were fixed parallel to each other at a distance of 0.15 m.

### 2.2. 3D Position and Motion Estimation

**Figure 1.**
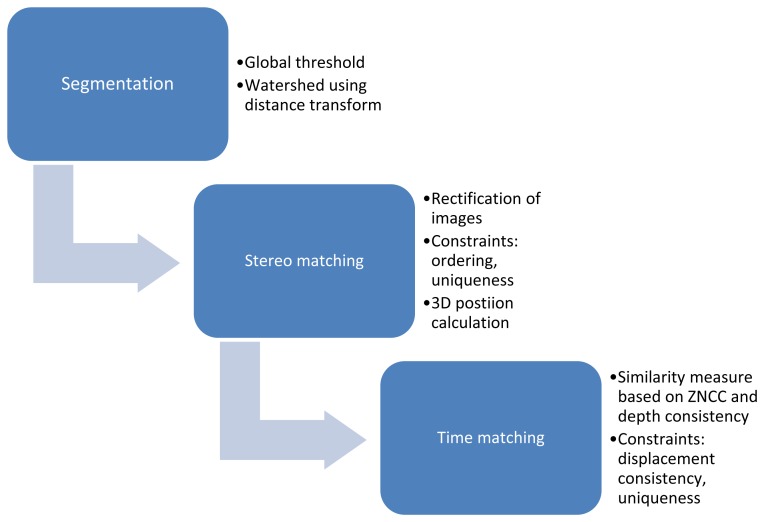
Steps of the 3D position and motion estimation algorithm.

An algorithm was developed to estimate parameters of fertilizer particles leaving the disk of a centrifugal fertilizer spreader. The aim was to accurately determine the 3D position and velocity of individual particles based on subsequent framesets of stereo images. The main steps of the position and motion estimation algorithm are illustrated on the following flowchart (Figure 1).

#### 2.2.1. Segmentation

**Figure 2.**
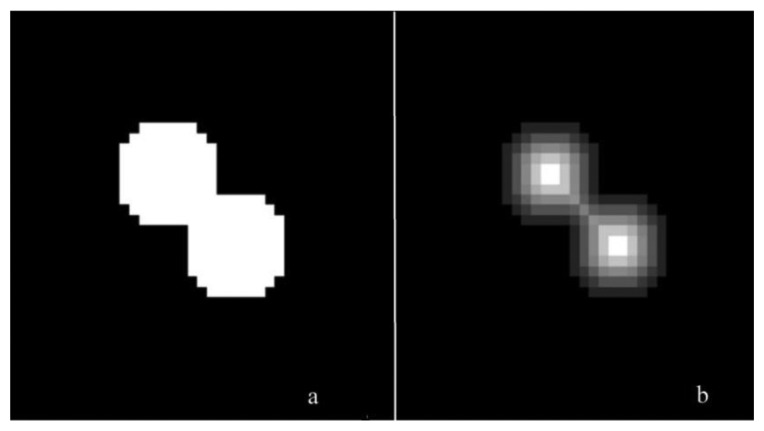
(**a**) Original image (**b**) Distance transformed image.

As a first step, image quality was enhanced and particles were segmented from the background. Histogram stretching increased contrast on the images. Next, a segmentation pass eliminated the background by applying a similarity operation. For application in the controlled environment, segmentation using a global threshold on the intensity values was sufficient. The connected components were determined and labelled. This allowed treating particles as entities instead of groups of individual pixels, which reduced computation time of following processing steps drastically. A second segmentation pass was used to reduce noise and separate overlapping particles. Objects with their minor axis length smaller than 3 pixels (approx. 2 mm) were treated as noise and were removed from the image. Objects with their major axis length larger than 13 pixels (approx. 9 mm) were presumed to consist of overlapping particles and were separated using a distance transform followed by a watershed segmentation. A distance transformation computes, on a binary image, the distance between each non-zero pixel and the nearest zero pixel. The effect is illustrated in Figure 2.

A discontinuity operation was used to separate overlapping particles: the watershed method [21]. The principle of this operation is simple. Images are considered to display a topographic relief. The gray level (monochrome cameras are used) of each point determines its height. Then, water is considered falling on the topography (image) and forms what is called catchment basins. The watersheds are thus the lines separating these basins.

#### 2.2.2. Stereo Matching and Calculation of 3D Position

The second part of the algorithm consists of a stereo matching step. The matching of particles between the images of the left and right camera is an important and critical stage to accurately determine the 3D position (and subsequently the 3D velocity) of the particles. Different stereo matching algorithms were evaluated by the framework developed in [22]. These were investigated to determine the most suitable and efficient one for this application. Direct application of one of these stereo matching algorithms was not possible. The first reason lies in the nature of the spreading process. Because the particles are not ejected in the same plane and the camera positions are different, the 2D projection of a throw on the image sensors differs. The possible difference between these patterns on the paired stereo images will give an erroneous stereo matching resulting in an incorrect 3D position. The second reason relates to the particles dimensions. Particle resolutions are low (particles have a bounding box between 3 × 3 and 13 × 13 pixels^2^ for the used setup) and the particles are very similar in shape and texture. This restricts the use of correlation techniques for finding stereo matches.

**Figure 3.**
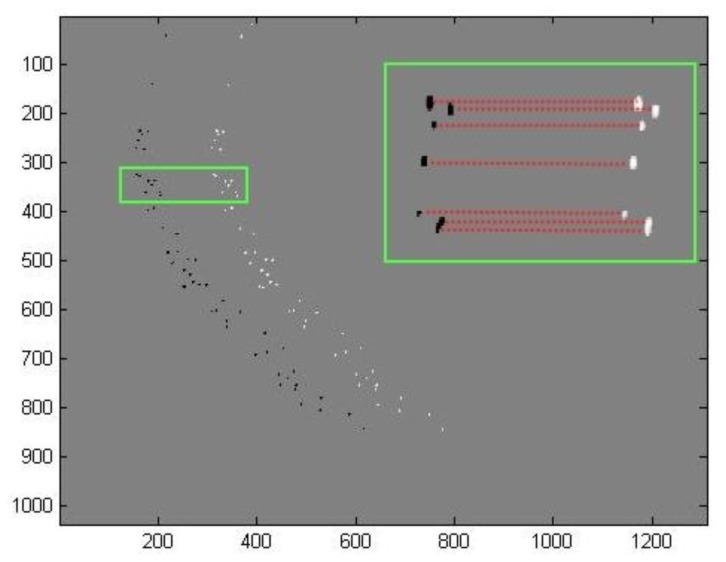
Illustration of stereo matching. For clarity, left and right images are superimposed. Black and white particles represent particles visualized by the right and left camera, respectively. The search for matches is done over horizontal scan lines.

Therefore a new algorithm was developed for stereo matching using rectified images. Based on the internal and external parameters, which were determined using machine vision software (Halcon version 2007, MVTech, München, Germany), a rectification map was created. This transformation map describes mapping of the images of the stereo camera to a common rectified image plane in a way that pairs of conjugate epipolar lines become collinear and parallel to the image axes. This means that the matching can be simplified to a one dimensional search along horizontal scan lines. It was assumed that the order of particles along horizontal lines was respected in the rectified stereo images (ordering constraint). The horizontal search is illustrated in Figure 3. Furthermore, instead of determining the match for each pixel of each particle, only particle centers were used for this operation. This considerably reduced computation time. Therefore, the 3D position of the center of every particle was determined. The center was determined as the center of gravity of the area of the apparent surface area of the particle on the images. Particle centers in the left image were matched with particles centers in the right image.

Particles with a match were removed from corresponding images to comply to the uniqueness constraint since each particle can only have one match. Finally the disparity between corresponding particles in both stereo images was calculated as the difference in column (pixel) number between both particle centers. In combination with the internal and external camera parameters, the disparity allows determination of the 3D position (*x,y,z*) of the particles relative to the left camera coordinate system:

(1) $$\{\begin{array}{c} x=\frac{x_{l}\;z}{f}\\ y=\frac{y_{l}\;z}{f}\\ z=\frac{fb}{d} \end{array}$$

with: (*x_l_*, *y_l_*) the position of the particle on the left image, d the disparity, b the translation between both cameras (all in m).

Coordinates are transformed to a world coordinate system (*x_w_*, *y_w_*, *z_w_*) using the following rigid transformation:

(2) $$\left[\begin{array}{c} x_{w}\\ y_{w}\\ z_{w}\\ 1 \end{array}\right]=\left[\begin{array}{cc} R & T\\ 0 & 1 \end{array}\right]\;\left[\begin{array}{c} x\\ y\\ z\\ 1 \end{array}\right]$$

where **R** the rotation matrix, **T** the translation matrix.

Particles without match were supposed half occluded and their disparity was considered equal to the mean disparity of the neighboring particles.

#### 2.2.3. Matching of Particles in Time

**Figure 4.**
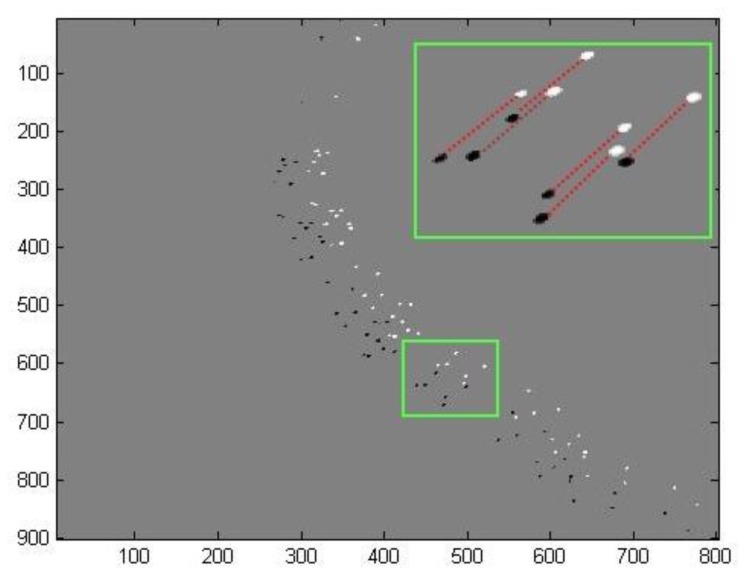
Illustration of matching particles in time. The estimated local displacements for some fertilizer particles are illustrated by red lines. For clarity, both images were superimposed.

At this point, the 3D position of fertilizer particles leaving a spreading disc can be automatically determined relative to the camera coordinate system. This was done by matching corresponding fertilizer particles on the stereo images of the high speed stereovision setup. However, also the 3D velocity of these particles should be known. Therefore images were taken at two subsequent time intervals *t*_0_ and *t*_1_. By matching fertilizer particles in the left image *I_l_*, the 2D displacement was calculated. Figure 4 illustrates the matching process. In combination with the used frame rate and the 3D position information, this enabled calculation of the particle velocity in three dimensions. To reduce computation time, a region of interest (ROI) was selected for further processing. This was done by selecting the region of the image containing all segmented particles.

The matching of particles in time implies that for every particle in the first image, the corresponding position in the subsequent image was determined. The matching step consisted of two steps: a global and a local motion estimation step. The global search was initiated by dividing the throws in subsequent left images into zones. For each zone, a global displacement vector was calculated by connecting the centers of gravity of the zones following their angular order. Afterwards, local motion estimation was performed based on a similarity factor. This factor was the sum of two terms. The first is a local correlation term based on Zero Mean Normalized Cross Correlation (ZNCC) similar to the one described in [19]. The degree to which two equally sized 2D patterns *(P*_1_, *P*_2_*)* are correlated was calculated as:

(3) $$R=\frac{\sum_{i}\sum_{j}({\text{P}}_{1}-\bar{{\text{P}}_{1}}).({\text{P}}_{2}-\bar{P_{2}})}{\sqrt{\sum_{i}\sum_{j}(P_{1}-\bar{P_{1}}).(P_{1}-\bar{P_{1}})}\sqrt{\sum_{i}\sum_{j}(P_{2}-\bar{P_{2}}).(P_{2}-\bar{P_{2}})}}$$

where i and j are the number of rows and resp. columns of patterns *P*_1_ *and P*_2_, and *R* is the correlation term (all dimensionless).

The depth consistency term improved the matching process because it takes into account the relative vertical position of the particles which will, at least in the beginning of the ballistic flight of the particles, not drastically change in time. The depth consistency term was calculated based on the depth ranking. For every particle in the first image, the depth relative to neighboring particles in a pattern centered around the particle was compared with the depth of possible candidate particles on the subsequent image relative to their neighboring particles. If the candidate particle had the same rank as the investigated particle, then the consistency was respected; if not, then a truncated linear model was used, which is given in Equation (4):

(4) $$V(d)=\{\begin{array}{c} 1-\frac{\left|d-d_{0}\right|}{d_{0}}if\left|d-d_{0}\right|<\rho \\ 0\;\text{otherwise} \end{array}$$

where *d* the relative depth of a candidate particle in the second image [mm], *d_0_* the relative depth of the investigated particle on the first image [mm], *ρ* a threshold value [mm] and V(*d*) is the consistency cost [-].

As mentioned before, the displacements of the centers of the segmented particles were used instead of the displacements of all the particle pixels to reduce the computation time. In addition, the candidates giving the second and the third best similarity measure were saved and sometimes used instead of the particle with the best similarity measure depending on the value of a consistency constraint which will be explained below.

The final step of the motion estimation algorithm aimed to improve the estimated displacement determined previously. To increase the accuracy, uniqueness and displacement consistency constraints were included in this part of the motion estimation algorithm as well. The first constraint assumed that in the first meter of traveling after the ejection, the displacement of a particle is close to the displacement of its neighbor. To enforce this, each estimated particle displacement was compared to the median of its neighbors displacement. The comparison was performed using the absolute difference (AD) in angle relative to the image coordinate system. If the AD was smaller than a threshold value, the estimated displacement was set consistent and the displacement is confirmed. In the other case, the displacements calculated with the second and the third best similarity measures were compared with the median displacement and the first one validating the consistency condition was approved. If the consistency constraint was not respected, the particle was transferred to a second motion estimation pass which is described below.

The second constraint is a uniqueness constraint. This means that each particle in the first image can only be mapped to maximum one particle in the second image and inversely one particle in the second image should be mapped to maximum one particle in the first image. The first part of this constraint was respected by choosing the particle that gives the best match for the similarity measurement. Nevertheless the second part was not considered and a particle in the second image could be mapped to multiple particles in the first image if these all give the best similarity factor. Therefore if multiple particles were mapped to one particle, their estimated displacements were not approved and they were set to a second motion estimation pass.

In the second motion estimation pass, only particles that did not respect the previous constraints were treated. New images containing these particles were created by eliminating mapped particles from both images. To improve the motion estimation, the median of the displacement vectors of neighboring particles replaced the global displacement vector.

### 2.3. Validation

#### 2.3.1. Stereo Matching Algorithm

To quantify the accuracy of the stereo matching algorithm, stereo reference images with a known disparity map should be used. However, an accurate reference disparity map of a real fertilizer spreading process was almost impossible to obtain. Therefore both simulated stereo images and their corresponding disparity map were generated by a simulator, consisting of two main parts: the particle position generator and the virtual stereo rig [23]. The position simulator modeled the 3D position and displacement of fertilizer particles leaving a spreading disk while the virtual stereo rig generated the stereo images and the resulting disparity map. The stereo matching algorithm was applied to the resulting stereo images and the resulting disparity map was compared with the disparity map generated by the spreading simulator. For validation, the comparison was performed on 20 sets of simulated stereo images. Each image pair contained all fertilizer particles of one throw. The segmentation was validated manually on all images.

#### 2.3.2. Position- and Motion Estimation

**Figure 5.**
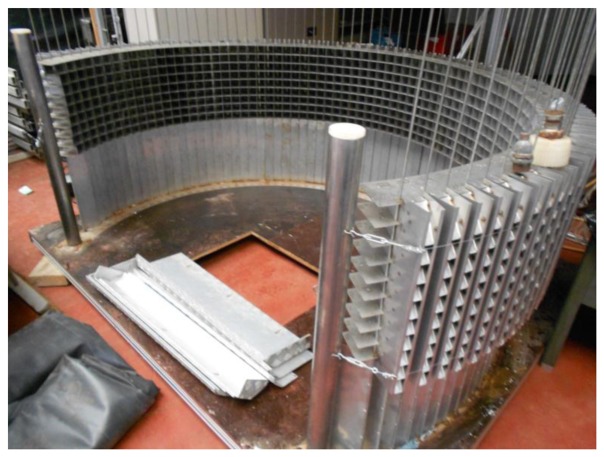
Cylindrical collector used for validation of the system.

The performance of the high speed binocular stereovision setup in combination with the position and motion estimation algorithm was validated experimentally. Because reference data for the positions and velocities of fertilizer particles could not be obtained in practice, the system was validated by measuring the cylindrical spread pattern. The cylindrical spread pattern is less influenced by uncertainties in the ballistic flight model and does not require a large hall to measure the distribution of fertilizer particles on the ground. The spread pattern was measured using a cylindrical collector with 78 compartments that were arranged around the center of the spreading disk to form a cylindrical area with a radius of 1 m. The collector is illustrated in Figure 5.

Each compartment corresponded with 3 degrees resulting in an arc of 234 degrees around the circumference of the spreading disk. Vertically, every compartment was divided in 9 sub-compartments with 0.05 m height. The orientations of the vertical edges were adjustable in order to minimize the collision between particles and the borders of the compartments. For one test, the spreader was activated during 30 s.

The collected particles in each sub-compartment were weighed and the horizontal and vertical angular (mass) distributions were calculated. The resulting cylindrical distribution was compared with a simulated distribution pattern which was calculated using the stereovision setup and corresponding image processing algorithms developed in this study. In total, 51 throws were analyzed. Every throw was captured on image two times (with a period of 0.001 s in between) to generate two subsequent sets of stereo images. The stereo matching algorithm led to an automated determination of the 3D position of fertilizer particles relative to the left camera coordinate system. The time matching algorithm calculated the velocity of the particles in three dimensions. Using both the position and velocity information in a ballistic flight model, the trajectory of each particle was simulated. Based on the intersection of the trajectory with a cylinder with similar geometry as the collector, the cylindrical distribution was calculated. Finally the measured and simulated distributions were compared. The results enabled validation of the performance of the high speed stereovision setup in combination with the 3D position and velocity estimation algorithms.

Two quantities were used to compare the measured and the predicted distributions of fertilizer: the 2D correlation coefficient *C*_1_ (Equation (5)) and the Relative Error *C*_1_ (Equation (6)):

(5) $$C_{1}=\frac{\sum_{m}\sum_{n}(A_{mn}-\bar{A})(B_{mn}-\bar{B})}{\sqrt{\left(\sum_{m}\sum_{n}{\left(A_{mn}-\bar{A}\right)}^{2})(\sum_{m}\sum_{n}{\left(B_{mn}-\bar{B}\right)}^{2}\right)}}$$

where *A_mn_* and *B_mn_* are the estimated and measured weight in the compartment in row m and column n, respectively, and Ā and B ¯ are their mean values:

(6) $$C_{2}=\frac{\sum_{m}\sum_{n}(A_{mn}-B_{mn})}{\left(\sum_{m}\sum_{n}(A_{mn}))+(\sum_{m}\sum_{n}(B_{mn})\right)}$$

## 3. Results and Discussion

### 3.1. Segmentation and Stereo Matching

Results indicated that only 3.1% of the fertilizer particles were over- or under segmented. This illustrates that the segmentation step of the algorithm performs well under the used conditions. The segmentation step was found sufficient for the controlled conditions used for the tests.

**Figure 6.**
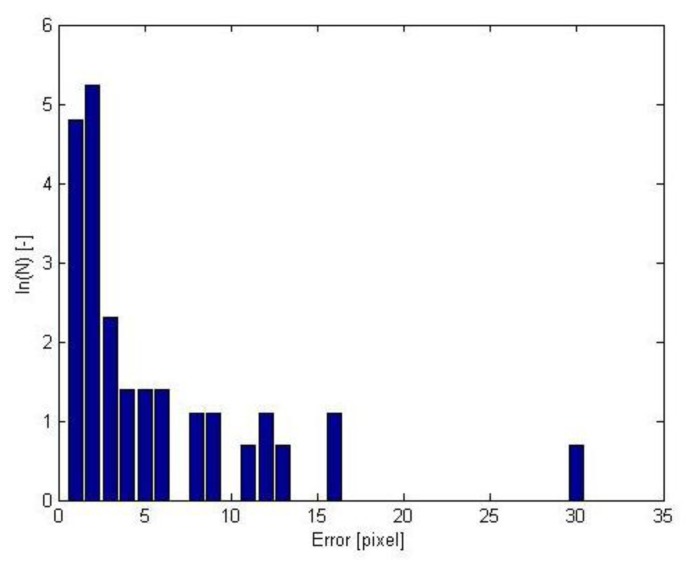
Histogram of the disparity errors of the particles: the natural logarithm of number of particles as a function of disparity error.

The simulated stereo images were used to validate the stereo matching algorithm. Figure 6 shows the histogram of the disparity errors. The natural logarithm was used to improve the readability of the occurrence of larger disparity errors. The proposed system showed accurate results: around 90% of the disparities were determined with an error of less than 2 pixels. The largest errors were mostly observed in regions of under-segmentation. These consist of a superposition of several particles that cannot be differentiated because of the low resolution of the cameras. This could be resolved using a higher resolution imaging system. It should, however, be noted that this drastically increases the cost of the high speed cameras which counteracts the use in practice.

### 3.2. Time Matching

**Figure 7.**
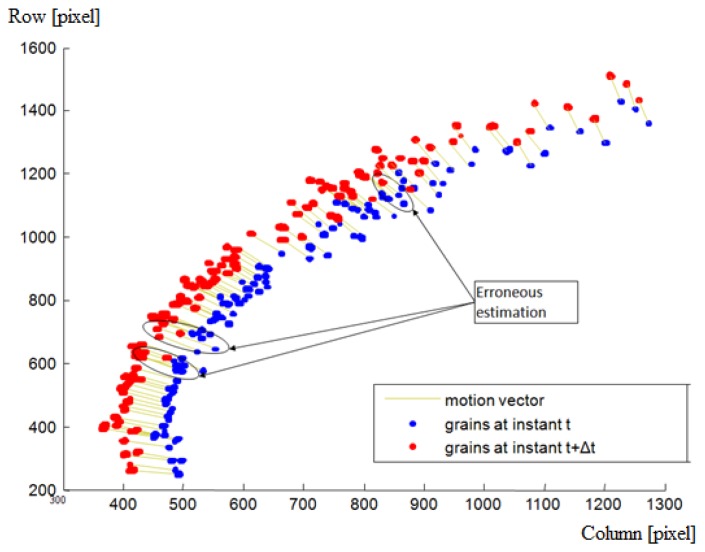
Estimated displacement after the first step of the motion estimation algorithm. The region of interest (bounding box of the throw) is illustrated in increased resolution.

**Figure 8.**
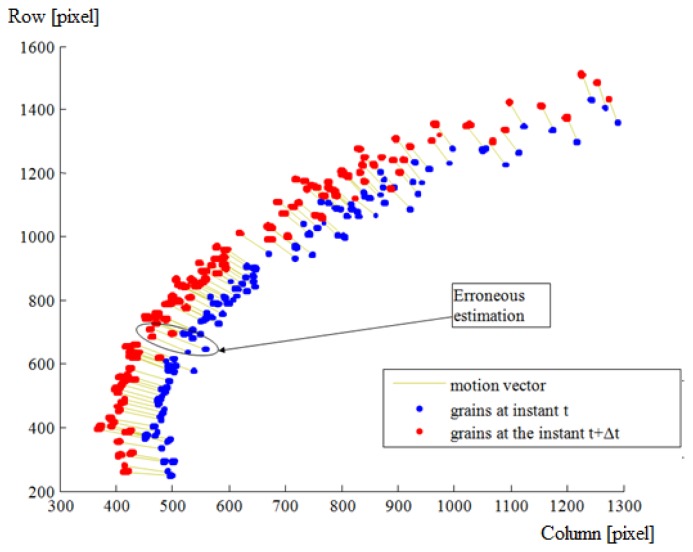
Corrected displacements based on the consistency and uniqueness constraint. The region of interest (bounding box of the throw) is illustrated in increased resolution.

As mentioned before, time matching was done in two main steps. The first step comprises a global estimation followed by a local estimation based on a similarity measurement. The second step corrects these estimations based on a consistency and uniqueness constraint. For one throw of fertilizer, Figures 7 and 8 illustrate the estimated displacements after these two steps respectively. The blue and red objects are the particle images at the instants t and t + Δt respectively. The estimated displacements are represented by lines connecting a given particle at the instant t to its supposed match at the instant t + Δt. The erroneous estimations are indicated as well. They were determined manually by detailed visual inspection.

### 3.3. Position and Motion Estimation

**Figure 9.**
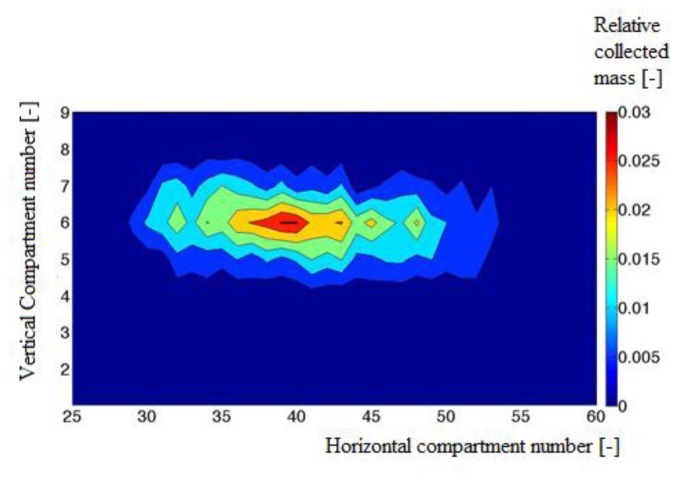
Experimentally determined cylindrical fertilizer distribution.

**Figure 10.**
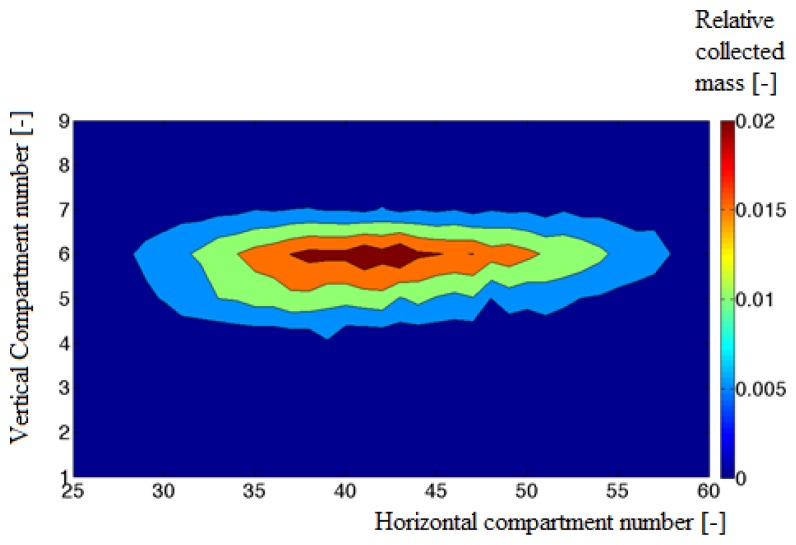
Cylindrical distribution simulated using the stereovision setup.

The performance of the hardware in combination with the image processing algorithms was validated by comparing the experimentally determined mass distribution (see Figure 9) with the simulated distribution (see Figure 10).

A correlation coefficient of 0.9 and a Relative Error of 27% were found. The mean quadratic error between the estimated and the measured horizontal and vertical distributions was found to be 9.1% and 2.2%. The simulated distribution showed local minima in comparison with the measured distribution. These local minima were caused by the small number of images due to the limited memory capacity of the cameras.

It should be noted that the simulated pattern is determined based on the number of particles in each compartment while the measured distribution, obtained from the collector is based on the total weight of all particles in a subcompartment. To have enough particles in all subcompartments of the collector the particles are spread during at least 2 s. Because of the limited memory of the high speed camera, the imaging system can only record the spreading process during a short time (namely 0.7 s) of the spreading experiment, corresponding to 14 throws at a disk speed of 600 rev/min and using two vanes. Therefore the number of collected particles in the subcompartment is much higher than the number of particles analyzed on the recorded images. To deal with these restrictions, the experiments were performed with particles of similar size (3.3–4 mm) obtained after sieving. Hence we can assume that the weight is nearly proportional to the number of particles. In addition, we assume that the spreading process is static which means that the distribution does not change when the spreader and particles characteristics stay the same.

To accurately determine the distribution of fertilizer particles on the ground, not only the position and velocity of the particles should be known, also the diameter, true density and shape should be known to accurately predict the individual landing positions and subsequently the spread pattern. Inaccuracies in these parameters can lead to a biased spread pattern even when the position and velocity of the particles were very precisely determined. Because shape and diameter can be determined using image processing, the algorithm can be extended to measure these parameters for individual particles. Because concurrent measurement of the true density is not possible, the variability in true density between particles of similar fertilizer types should be investigated. Also, the variability in mass flow leaving the hopper of the fertilizer spreader should be determined. Furthermore, spinning of particles has to be studied and included in the ballistic flight model because this can have a considerable effect on the landing position [24].

## 4. Conclusions

In this paper we proposed a new 3D imaging system for characterization of parameters of fertilizer particles leaving the disk of a centrifugal fertilizer spreader. The system combines a high speed binocular stereovision image acquisition system with a 3D position and motion estimation algorithm. The algorithm consisted of a segmentation, stereo matching- time matching algorithm. Segmentation was done based on a global threshold and a discontinuity operator to separate overlapping particles. In addition, particles were clustered to reduce computation time. Stereo matching was performed over horizontal scan lines since images were rectified. The motion estimation algorithm was based on two steps. The first step was a similarity measurement based on local correlation and a depth consistency term. The second step corrects the estimated displacements based on a consistency and uniqueness constraint.

The segmentation and stereo matching algorithm were validated using a 3D stereovision simulator for centrifugal fertilizer spreading. Accurate results were obtained: about 90% of the disparities were determined with an error of less than 2 pixels.

The performance of the setup in combination with developed algorithms was validated using an experimental spreader and cylindrical collector. The position and velocity of individual fertilizer particles leaving the disk were determined and were used in combination with a ballistic flight model to simulate the cylindrical distribution pattern. By comparing this with the measured cylindrical distribution, the accuracy of the system was determined. A 2D correlation coefficient of 90% and a Relative Error of 27% were found between the experimental results and the (simulated) spread pattern obtained with the developed system.

In the current state, the developed system is not suitable for a field application yet. The system can only be used under controlled conditions because of the high cost and fragility of the high speed cameras. Therefore an alternative 3D high speed imaging system based on multi-exposure is under investigation. Because it is based on standard cameras, this system will offer higher resolution stereo images at a significantly lower cost. In order to be able to combine it with the algorithms developed in this work, several image pre-processing features should be added such as splitting the images to extract particles in time. Also the interference of the (variable) daylight and various background features in a field application should be studied. The adaptation will make the system considerably more cost efficient and has potential application as online feedback sensor on modern fertilizer spreaders.

It can be concluded that the 3D technique developed in this study can be used to perform spreading characterization in controlled conditions. This can be used for spreader design for example to evaluate the effect of different spreader settings. Future research will focus on the investigation of the true density variability within fertilizer type and the variability in mass flow leaving the hopper of fertilizer spreaders. The algorithms developed in this work will be extended with an algorithm to determine the diameter and shape of individual particles. In combination with a more complex ballistic flight model, this will enable calculating the spread pattern on the ground. This can be used as a tool for manufacturers to directly inspect the effect of spreaders settings on the spread pattern, to enable an affordable spreader calibration at farm level and as online feedback sensor for modern fertilizer spreaders.
